# Inhibition of lipolysis in visceral adipose tissue from obese mice and humans prevents impairment of endothelial Kir2.1 channels

**DOI:** 10.1080/19336950.2025.2564651

**Published:** 2025-09-25

**Authors:** Emma C. Hudgins, Erica J. Johnson, Sabita Rokka, Bhaswati Kashyap, Arielle Mahugu, Thanh Nguyen, Anthony R. Tascone, Elizabeth McCarthy, Caitlin Halbert, Ibra S. Fancher

**Affiliations:** a Department of Kinesiology and Applied Physiology, College of Health Sciences, University of Delaware, Newark, DE, USA; b Department of Physiology and Biomedical Engineering, Mayo Clinic, Rochester, MN, USA; c Christiana Care Health System, Department of Surgery, Section of Bariatric Surgery, Wilmington, DE, USA

**Keywords:** Obesity, visceral adipose, Kir2.1 channels, fatty acids, endothelial dysfunction

## Abstract

Accumulation of abdominal visceral adipose tissue (VAT) is a major risk factor for cardiovascular disease. Obesity-induced endothelial dysfunction is a precursor to severe disease, and we and others have shown that arteries embedded in VAT, but not subcutaneous adipose tissue, exhibit robust endothelial dysfunction. Using a mouse model of diet-induced obesity, we recently linked VAT from obese mice to the impairment of endothelial Kir2.1, a critical regulator of endothelial function. However, the mechanism by which VAT impairs Kir2.1 is unclear. As Kir2.1 impairment is dependent on endothelial CD36, we hypothesized that lipolytic VAT induces Kir2.1 impairment through fatty acids (FA). To test this, we first treated endothelial cells with palmitic acid (PA) to determine whether the addition of exogenous FAs recapitulated our original finding of Kir2.1 dysfunction when challenged with VAT. PA inhibited Kir2.1 assessed via whole-cell patch-clamp electrophysiology, an effect that was dependent on endothelial CD36. To determine whether inhibiting VAT lipolysis prevents Kir2.1 dysfunction in the presence of VAT in obese mice and humans, VAT was pretreated with small molecule inhibitors of adipose triglyceride lipase prior to incubating endothelial cells with adipose tissue. This approach also prevented VAT-induced impairment of endothelial Kir2.1 suggesting that VAT-derived FAs may play a role. Furthermore, inhibition of lipolysis in the VAT of obese mice and humans significantly reduced endothelial FA uptake, similar to that observed when CD36 was downregulated. These findings advance our understanding of the relationship between VAT and endothelial Kir2.1 impairment and place VAT-derived FAs as potential paracrine mediators.

## NEW AND NOTEWORTHY

These findings advance our understanding of the role of VAT in mediating endothelial cell dysfunction by highlighting the impact of adipose lipolysis and FAs on Kir2.1 channels in obesity. The present findings, generated from a mouse model of diet-induced obesity and adipose tissue from humans with obesity, suggest that VAT-derived FA may promote endothelial Kir2.1 dysfunction, perhaps following elevated FA uptake, as an underlying mechanism governing obesity-induced endothelial dysfunction.

## Introduction

The accumulation of abdominal visceral adipose tissue (VAT), which underlies visceral obesity, is strongly correlated with insulin resistance, diabetes, and cardiovascular disease risk [1–5]. In contrast to subcutaneous adipose tissue (SAT), VAT exhibits an increased inflammatory profile, accompanied by altered metabolic efficiency [6–11]. VAT is also a major contributor to elevated circulating free fatty acids (FAs) and, therefore, insulin resistance under obesogenic conditions [7,12–14]. Furthermore, we and others have shown that arteries located in the VAT, but not SAT, exhibit robust endothelial dysfunction, a well-established precursor to cardiovascular disease that can also arise from increased FA [15–18]. Taken together, VAT represents a major driver of the development of insulin resistance, endothelial dysfunction, and cardiovascular disease associated with visceral obesity.

We recently showed that VAT from obese mice impairs endothelial inwardly rectifying K^+^ 2.1 channel (Kir2.1) function in vitro, whereas VAT from lean mice and SAT from lean or obese mice had no effect [19]. Kir2.1 channels are critical regulators of endothelial function and have been shown to contribute to nitric oxide production, endothelium-dependent hyperpolarization of smooth muscle, and angiogenesis [20–22]. We previously established a dichotomy in Kir2.1 function in the VAT vs. SAT artery endothelium in a mouse model of diet-induced obesity and in humans with obesity [16]. Specifically, Kir2.1 was robustly dysfunctional in the VAT artery endothelium but retained function in the SAT artery endothelium. Next, we revealed that Kir2.1 function was restored in endothelial cells with reduced expression of the scavenger receptor CD36, an established FA-translocase affiliated with cardiovascular disease [23], and in the VAT artery endothelium of obese CD36 knockout mice, suggesting that VAT inhibits Kir2.1 through CD36 [19]. These findings support a potential paracrine role for VAT in mediating endothelial dysfunction through the impairment of Kir2.1; however, the link between VAT and endothelial CD36 upstream of Kir2.1 impairment is yet to be established.

Based on i) our previous findings detailing a critical role for CD36 in the impairment of Kir2.1 in response to VAT in obese mice [19]; ii) the ability of CD36 to efficiently mediate FA uptake [24], and iii) the putative capacity of FA derivatives to inhibit endothelial Kir2.1 [25,26], we first hypothesized that exogenous addition of the common FA palmitic acid (PA) impairs Kir2.1 function in a CD36-dependent fashion, similar to our observations where endothelial cells were challenged with VAT from obese mice. Next, based on the role of VAT in increasing levels of circulating FAs in obesity [12–14], we predicted that inhibiting lipolysis in VAT from obese mice and humans ex vivo would restore endothelial Kir2.1 function and prevent the VAT-mediated increase in FA uptake, again replicating effects that were previously observed when endothelial CD36 was downregulated.

## Materials and methods

The authors confirm that this study adheres to the ARRIVE guidelines.

### Animals

All animal studies were approved by the Institutional Animal Care and Use Committee of the University of Delaware under protocol #1372–2023. Nine-week-old C57BL/6J WT mice were purchased from The Jackson Laboratory (WT stock no. 000664) and housed in the same room under 12 hour light-dark cycles at the University of Delaware Life Sciences Research Facility animal vivarium. Mice were housed five mice per cage which contained *Bed-o-Cob* bedding and *Enrich-n’Nest* paper roll enrichment. After one week of acclimation, 10-week-old mice were randomly divided into diet groups: lean controls maintained on a normal laboratory diet and an obese group fed a high-fat Western diet (42% kcal from fat) (Envigo-Inotiv; Cat. No. TD.88137) commonly used to induce obesity in mice [6,16,19]. The respective diets were provided ad libitum and were maintained for 8–10 weeks, a time point at which obvious weight gain was observed (Table S1), and robust endothelial dysfunction was observed in WT mice [16,27]. The mice were euthanized by cervical dislocation followed by thoracotomy. SAT from the abdominal and hindlimb regions and mesenteric VAT were isolated for use in in vitro studies. VAT and SAT were processed in HEPES buffer and used to treat the cells on the same day of dissection. To obtain the desired amount of adipose for these studies, fine forceps and microscissors were used to gently separate small amounts of tissue from the gross dissected adipose, using caution to avoid extensive mechanical perturbation that may influence FA release. The separated tissue was lightly dried with a Kim wipe to remove excess HEPES buffer and then placed on a microbalance until 10 mg dry weight was collected for immediate use in downstream applications. Endothelial cells in culture were exposed to adipose collected this way within one hour of the animal dissection. A total of 60 mice, with equal numbers of male and female mice, were used in this study. No significant sex differences were found; therefore, the data were combined. Each animal used contributed to a given data set without cause for removal from the study.

### Human studies

Human studies were conducted with prior approval from the Institutional Review Board of the Christian Care Health System (CCHS) in collaboration with surgeons in the Christian Care Bariatric Surgery Program (Christiana Care Corporation Approval no. 42036). Studies involving human participants were performed in accordance with the guidelines stated in the Declaration of Helsinki. Participants recruited for this study provided written informed consent prior to the date of the planned bariatric surgery. Subjects (*n* = 4) had body mass indices ranging from 44.9 to 50.3 and were between 29 and 47 years old. Subjects were excluded from the study if they had cancer, heart disease, history of smoking, kidney or liver disease, gallbladder disease, or autoimmune/inflammatory disease. Participants were also excluded if they had hypertension or hypercholesterolemia that was not controlled by medication, as these risk factors have been shown to impair endothelial Kir channels [28,29]. The patient medications are listed in Supplemental Table S2. All subject information was de-identified and stored on a password-protected, on-site hard drive at the CCHS. Approximately 1 g each of abdominal SAT and VAT (omental) adipose biopsies was collected during surgery by a bariatric surgeon under the IRB protocol. A member of the University of Delaware lab retrieved the biopsies on the day of the surgery and stored the tissue as ~50 mg aliquots in liquid nitrogen until use. Adipose retrieved from liquid nitrogen was allowed to thaw in chilled HEPES before obtaining 10 mg dry weight for downstream applications as described above.

### Cell culture

Human adipose microvascular endothelial cells (HAMECs) were maintained under standard culture conditions using an endothelial cell medium and supplements (ScienCell). Low-passage cells between P2-P4 were used in all experiments. Cells were seeded at ~ 80% confluency and allowed to form a monolayer prior to en bloc treatment with adipose tissue (5 mg/ml) from lean or obese mice or humans with obesity, as previously described [19]. The cells were incubated with adipose tissue for 48 h under standard culture conditions prior to downstream analysis. Untreated cells were used as controls. To inhibit adipose tissue lipolysis in VAT or SAT isolated from mice, adipose tissue was incubated with 20 μM Atglistatin (Cayman Chemical) or 0.002% DMSO (vehicle control) for 2 h in medium at 37°C prior to treatment. To inhibit adipose tissue lipolysis in VAT or SAT from human subjects, adipose tissue was incubated with 500 nM NG-497 (Cayman Chemical) or 0.0005% DMSO (vehicle control) for 2 h in medium at 37°C prior to treatment. To determine the effects of CD36 downregulation in vitro, HAMECs at 50% confluency were transfected with siRNA against CD36 (CD36 Silencer® Select from ThermoFisher Scientific, Cat. No. 4392420) or scrambled siRNA control (Silencer™ Select Negative Control from ThermoFisher Scientific, Cat. No. 4390843) using Lipofectamine (RNAiMax, Invitrogen), following the manufacturer’s instructions, prior to treating cells with PA.

### Patch clamp electrophysiology

After treating the cells with PA, they were washed 2x with RT PBS. Following adipose tissue treatment, the tissue was discarded, and cells were washed 2x with RT PBS. HAMECs were then non-enzymatically dissociated using Versene (Gibco), resuspended in bath solution, and stored on ice. A whole-cell voltage clamp was performed on the HAMECs using thick-walled borosilicate glass pipettes (Sutter Instrument) with resistances of 3–5 MΩ after fire polishing. Currents were recorded in a high 60 mM K^+^ bath to detect inwardly rectifying K^+^ currents using an EPC10 amplifier (HEKA Electronik) and the accompanying acquisition and analysis software (Pulse and PulseFit). HAMECs were held at −30 mV and a voltage ramp of − 140 to +40 mV was applied over 400 ms. Currents were low-pass filtered at 2 kHz and recordings were digitized at 10 kHz. Prior to collecting data for analysis, the inwardly rectifying K^+^ currents were stabilized. For recordings to be accepted for offline analysis, the patch needed to maintain a stable membrane resistance and series resistance ≤10 MΩ for the duration of the voltage ramp protocol, which consisted of at least 20 stable recordings in a static bath. The currents were normalized to the cell capacitance to obtain the current densities (pA/pF) for analysis. When necessary, leak subtraction was performed offline to collect the most accurate data points at −100 mV for group analyses.

### Immunocytochemistry

Following adipose tissue treatment, cells were washed 2x with RT PBS and then fixed at room temperature without permeabilization for 15 min using 4% paraformaldehyde (PFA) in PBS. After fixation, heat-mediated epitope retrieval was performed at 95°C for 5 min in antigen retrieval buffer (Tris-EDTA, pH 9.0). The samples were removed from the heat for 5 min, and the process was repeated twice. Antigen retrieval buffer was aspirated, and the cells were blocked for one hour at RT using 5% BSA in PBS on a slow rocker. Following blocking, cells were washed twice with PBS for 10 min. Primary antibodies targeting extracellular epitopes of CD36 (R&D Systems, polyclonal goat anti-mouse CD36, Cat. No. AF2519) or Kir2.1 (Abcam, rabbit monoclonal; Cat. No. AB109750) was diluted 1:100 in PBS containing 1% BSA. The cells were incubated with primary antibodies overnight at 4°C in the dark, with gentle agitation on a rocker. The cells were washed 2x with RT PBS for 5 min at room temperature with gentle rocking. The cells were then incubated with the corresponding secondary antibodies (donkey anti-goat Alexa Fluor 488 for detecting CD36 and goat anti-rabbit Alexa Fluor 647 for detecting Kir2.1) diluted 1:100 in PBS with 1% BSA for 1 h at room temperature in the dark. The cells were washed 2x again with RT PBS for 10 min. The cells were stained with DAPI and bathed in PBS. Immunofluorescence images were captured at 10x magnification using an EVOS fluorescent microscope, and at least three different fields of view/sample were imaged for analysis. Following background subtraction, the mean fluorescence intensity (MFI) of each image was measured by a blinded investigator using ImageJ [30] which was then normalized to the number of cells in the field of view determined by counting the DAPI-stained nuclei. An average MFI/sample was calculated using each field of view, which was then normalized to control, untreated cells similarly processed in each independent experiment. Each experiment also contained antibody control samples that were incubated with 1% BSA in PBS without the primary antibody. To further confirm the specificity of the primary antibodies for immunocytochemistry (ICC), siRNA against each target gene was used (CD36 Silencer® Select from ThermoFisher Scientific, Cat. No. 4392420; KCNJ2 Silencer® Select from ThermoFisher Scientific, Cat. No. AM167708).

### Fatty acid uptake

A QBT fatty acid uptake kit (Molecular Devices) was used to determine fatty acid uptake in vitro as previously described [19,31]. The kit uses a BODIPY-fluorescent dodecanoic acid analog previously validated as a suitable substrate for fatty acid transporters, similar to naturally occurring fatty acids, and a proprietary quenching agent [31]. Following adipose tissue treatment, the tissue was discarded and HAMECs were washed 2x with RT PBS. The cells were serum-starved for one hour prior to incubation with fluorescent fatty acids for 90 min at 37°C. The cells were then washed and bathed in fresh PBS for imaging using an EVOS fluorescent microscope. Live cells were imaged at 10x magnification with three-six fields of view per sample collected for analysis. Following background subtraction, the MFI of the entire field of view was measured by a blinded investigator in Image J [30], normalized to the cell number determined in bright field, and each field of view/sample averaged to obtain a final MFI value for each sample. The experimental values were normalized to the MFI of the control, and untreated cells were similarly processed within each independent experiment.

### Solutions and reagents

Atglistatin (Cat. No. 15284) and NG-497 (Cat. no. 368865) was purchased from Cayman Chemical Co., Ltd. Concentrated stock solutions dissolved in DMSO (Sigma) were serially diluted in culture media before treating the adipose tissue with the desired working concentration. The 6:1 PA:BSA conjugate was purchased from Cayman Chemical (Cat. No. 29558) and diluted in endothelial cell medium to a final concentration of 250 μM. A vehicle control stock was prepared at 0.8 mM BSA and 150 mM NaCl and diluted to final concentrations of 40 μM BSA and 7.5 mM NaCl. The bath solution for whole-cell patch clamp experiments contained (80 mM) 80 NaCl, 60 mM KCl, 10 mM HEPES, 1 mM MgCl_2_, 2 mM CaCl_2_, and 10 mM glucose, pH 7.4. The pipette solution contained 5 mM NaCl, 135 mM KCl, 5 mM EGTA, 1 mM MgCl2, 5 mM glucose, and 10 mM HEPES (pH 7.2). The HEPES buffer contained 145 mM NaCl, 5 mM KCl, 2 mM CaCl_2_, 1 mM MgCl_2_, 10 mM glucose, and 10 mM HEPES (pH 7.4).

### Statistical analyses

Sample sizes in the present study are in line with our recent observations using similar outcome approaches [19]. Data were normalized to untreated control cells. Nonparametric data sets were tested for significant differences using the Mann – Whitney U test when comparing the two groups. The Kruskal – Wallis test was followed by Dunn’s post-hoc test to compare multiple groups. Initial tests were set to *p* < 0.05 before post hoc corrections.

## Results

### Inhibition of lipolysis in VAT from obese mice and humans prevents VAT-mediated impairment of endothelial Kir2.1 function

**Figure 1.**
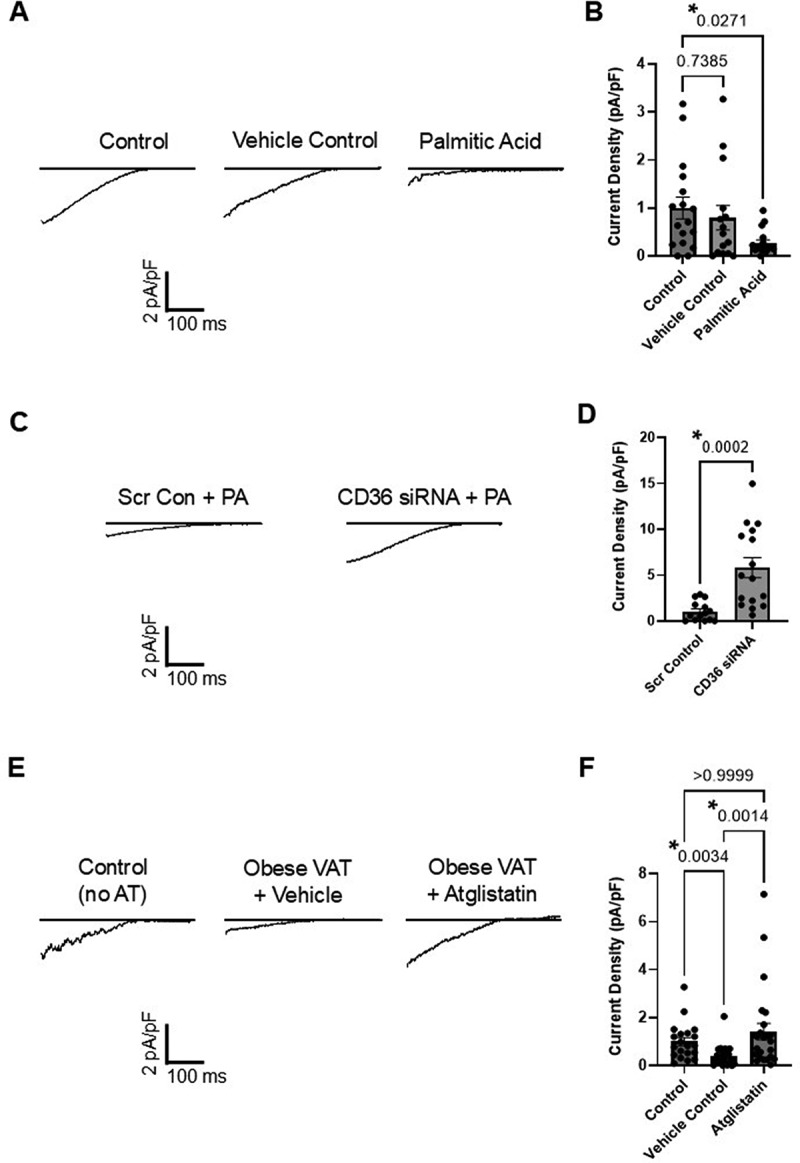
Inhibition of lipolysis in VAT of obese mice prevents impairment of endothelial Kir2.1. Human adipose microvascular endothelial cells (HAMECs) were incubated with either 200 μM 6:1 palmitic acid (PA):BSA or adipose tissue from lean or obese mice (en bloc; 5 mg/ml) for 48 h before assessing inwardly rectifying K^+^ currents via whole cell patch clamp. The holding potential was −30 mV, and the voltage ramp protocol was from − 140 to +40 mV over 400 ms. The representative recordings shown in A, C, and E reveal that the inwardly rectifying K^+^ currents reverse near the predicted reversal potential of approximately −21 mV under the specified patch conditions (i.e. 60 mM K^+^ bath, 140 mM K^+^ pipette solutions). A) Representative current recordings from untreated control HAMECs and HAMECs treated with either vehicle control (FA-free BSA) or PA. B) Group data show normalized current densities (pA/pF) analyzed and compared at −100 mV to determine the effects of PA treatment on Kir2.1 function (*n* = 15–17 cells/group tested over three independent experiments). Significant differences (*) were determined by a Kruskal-Wallis test (*p* = 0.0328) followed by Dunn’s post hoc tests. There was no difference in cell capacitance among groups (control, 46.5 ± 3.4 pF; vehicle, 46.0 ± 2.7 pF; FA, 52.2 pF ±4.4 pF; *p* = 0.5487). C) Representative current recordings from HAMECs treated with PA following pretreatment of cells with scrambled control (scr con) or CD36 siRNA. D) Group data show normalized current densities (pA/pF) analyzed and compared at −100 mV to determine the effects of CD36 downregulation on Kir2.1 function in the presence of PA (*n* = 14–16 cells/group tested over 3 independent experiments). A significant difference (*) relative to the scrambled control (scr con) was determined using a Mann-Whitney test. There was no difference in cell capacitance between groups (scrambled control, 64.8 ± 4.8 pF; CD36 siRNA, 62.3 ± 4.8 pF; *p* = 0.8773). E) Representative current recordings from untreated control cells (no AT) or cells exposed to VAT from obese mice pretreated with either DMSO vehicle (vehicle) or 20 μM Atglistatin. F) Group data reveal normalized current densities (pA/pF) that were analyzed and compared at −100 mV to determine the effects of treating VAT with Atglistatin on endothelial Kir2.1 function (*n* = 20–25 cells/group). VAT from four mice (two males, two females) was used across four independent experiments. Significant differences (*) were determined by a Kruskal-Wallis test (*p* = 0.0004) followed by Dunn’s post hoc tests. There was no difference in cell capacitance among groups (control, 56.6 pF ±4.8; vehicle, 51.8 ± 4.5 pF; Atglistatin, 60.7 ± 4.0 pF; *p* = 0.2649).

Our recent study revealed that VAT from obese mice inhibits endothelial Kir2.1 function in a CD36-dependent manner, suggesting the VAT/CD36/Kir2.1 axis is a potential contributor to obesity-induced endothelial dysfunction [19]. In the present study, we hypothesized that VAT-derived FA are responsible for Kir2.1 dysfunction based on the following lines of evidence: i) VAT lipolysis is a major contributor to circulating levels of FAs in obesity [12–14]; ii) CD36 is a well-established FA translocase that appears to be required for Kir2.1 impairment under obesogenic conditions [19,23]; and iii) Kir2.1 has been shown to be inhibited by internal FA derivatives [25,26]. Indeed, both the impairment of Kir2.1 and excess FAs have been linked to obesity-induced endothelial dysfunction [15,16,19,27]; however, no study to date has determined whether FA-mediated impairment of Kir2.1 serves as a potential mechanism. Therefore, our first goal was to determine if FAs inhibit Kir2.1 in a CD36-dependent manner similar to our observations in which endothelial cells were challenged by VAT from obese mice. Following 48 hr incubations with a pathophysiological PA:BSA ratio of 6:1, endothelial Kir currents were subsequently recorded via whole-cell patch clamp electrophysiology, as previously described [19]. Briefly, endothelial inwardly rectifying K^+^ currents, previously established to be largely conducted by Kir2.1 in human and murine adipose endothelial cells [21,27], were recorded using a voltage ramp protocol of −140 to +40 mV over 400 ms in a 60 mM K^+^ bath (representative recordings are shown in Figure 1(A)). Cells exposed to PA exhibited significantly reduced Kir current density compared to untreated control cells, while cells treated with FA-free BSA (vehicle control) showed similar average Kir current densities to the untreated control (Figure 1(A,B)). Furthermore, downregulation of endothelial CD36 using siRNA rescued the Kir current density in cells exposed to PA (Figure 1(C,D)). These findings are in line with our recent observations in endothelial cells challenged by VAT from obese mice and offer initial evidence that FA may underlie VAT-mediated suppression of Kir2.1 following CD36-mediated internalization of FA.

Our recent study also revealed that VAT from obese mice, but not VAT from lean mice or SAT from lean or obese mice, impairs endothelial Kir2.1 [19]. Based on the well-known role of VAT in increasing levels of circulating FAs in obesity and the putative capacity of FAs to inhibit Kir2.1, we next aimed to determine whether inhibiting lipolysis in VAT isolated from obese mice could prevent VAT-mediated impairment of endothelial Kir2.1. This was accomplished by treating isolated VAT with Atglistatin (20 μM), a specific small-molecule inhibitor of adipose triglyceride lipase (ATGL) [32], the rate-limiting enzyme in lipolysis, for 2 h prior to incubating endothelial cells with VAT (5 mg/ml). The selected dose of 20 μM was previously shown to significantly blunt FA cleavage from glycerol through the inhibition of ATGL activity in isolated adipose tissue similarly treated as in the present study [32]. Cells exposed to VAT treated with vehicle DMSO and untreated cells (no exposure to VAT) served as controls. Following 48 hr incubations with VAT from obese mice with or without Atglistatin treatment, endothelial Kir currents were again recorded via whole-cell patch-clamp electrophysiology (representative recordings are shown in Figure 1(E)). Inhibition of VAT ATGL with Atglistatin resulted in a significant restoration of endothelial Kir current density as compared to cells exposed to vehicle-treated VAT and reached similar average current densities observed in untreated control cells (Figure 1(E,F)).

**Figure 2.**
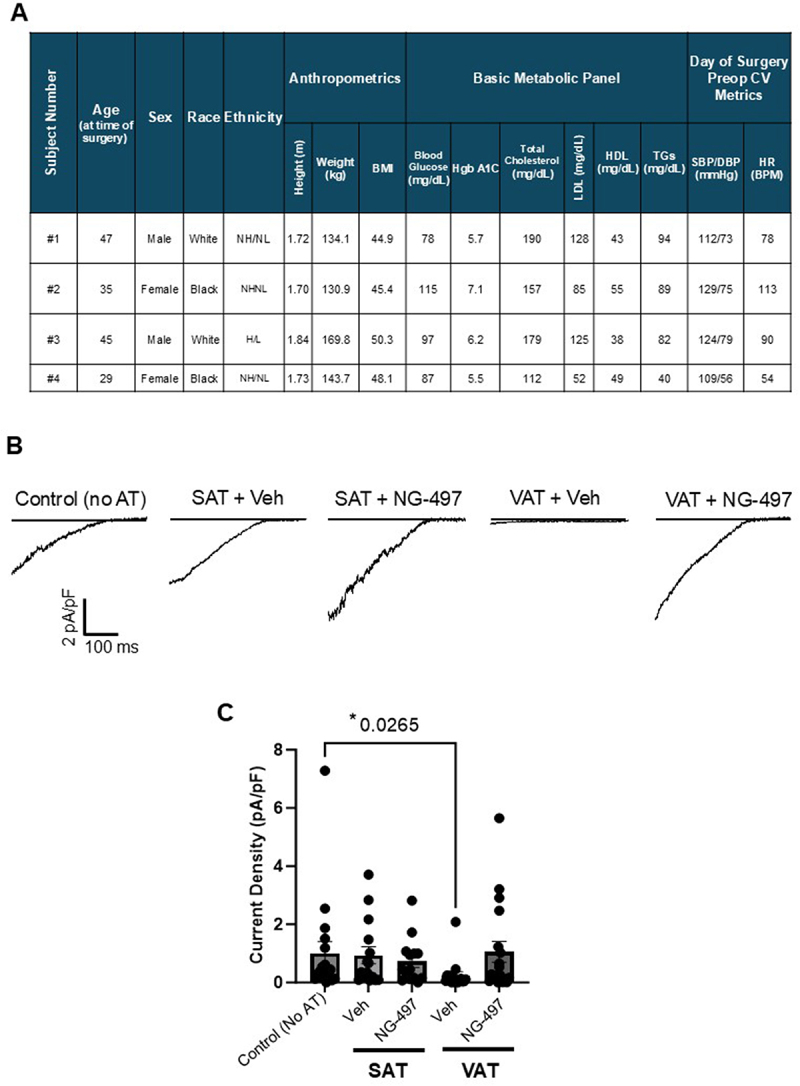
Inhibition of lipolysis in VAT from humans with obesity prevents impairment of endothelial Kir2.1. human adipose microvascular endothelial cells (HAMECs) were incubated with adipose tissue from human subjects with obesity (en bloc; 5 mg/ml) for 48 h before assessing inwardly rectifying K^+^ currents via whole cell patch clamp. The holding potential was −30 mV, and the voltage ramp protocol was from − 140 to +40 mV over 400 ms. The representative recordings shown in B reveal that the inwardly rectifying K^+^ currents reverse near the predicted reversal potential of approximately −21 mV under the specified patch conditions (i.e. 60 mM K^+^ bath, 140 mM K^+^ pipette solutions). A) the table details demographic, anthropometric, and clinical data from four subjects that provided adipose tissue to this study. Not Hispanic/not latino (NH/NL); Hispanic/latino (H/L); body mass index (BMI); low density lipoprotein (LDL); high density lipoprotein (HDL); triglycerides (TGs); systolic blood pressure/diastolic blood pressure (SBP/DBP); cardiovascular (CV); Heart rate (HR); beats per minute (BPM). B) representative current recordings from untreated control HAMECs (no AT) and cells exposed to either SAT or VAT from human subjects pretreated with either DMSO vehicle (vehicle) or 500 nM NG-497. C) group data reveal normalized current densities (pA/pF) that were analyzed and compared to untreated control cells at −100 mV to determine the effects of treating SAT and VAT with NG-497 on endothelial Kir2.1 function (*n* = 14–18 cells/group). Significant differences (*) were determined by a Kruskal-Wallis test (*p* = 0.0314) followed by Dunn’s post hoc tests. There was no difference in cell capacitance among groups (control, 36.4 ± 3.4 pF; SAT + vehicle, 40.4 ± 2.9 pF; SAT + NG-497, 38.0 ± 2.8 pF; VAT + vehicle, 41.5 ± 2.6 pF; VAT + NG-497, 37.9 ± 3.8 pF; *p* = 0.4817).

To determine whether these findings translate to human obesity, we incubated endothelial cells with VAT or SAT collected from individuals undergoing bariatric surgery. Figure 2(A) shows the demographic, anthropometric, and clinical data of the four subjects who provided adipose tissue for this study. Participants were between 29 and 47 years old, with an average BMI of 47.2, indicative of Class 3 obesity. As our goal was to determine the effects of adipose tissue on endothelial cell function with obesity as a major independent cardiovascular risk factor, the included subjects exhibited what is considered a normal to pre-risk range regarding parameters of a metabolic panel (i.e. blood glucose, Hb1Ac, total cholesterol, LDL/HDL, and triglycerides) and day-of-surgery blood pressure and heart rate. Only one subject in the cohort exhibited an Hb1Ac level indicative of diabetes (i.e. subject #2 at 7.1) and was therefore taking relevant medication (Table S2). We previously showed that endothelial Kir2.1 function was completely abrogated in arteries isolated from the VAT of obese humans, whereas arteries from SAT of the same subjects exhibited functional Kir2.1. Therefore, in the absence of true lean, “healthy” participants, SAT was used as an additional control to untreated cells, as we did not anticipate effects on Kir21 function based on past evidence [16,19]. VAT or SAT (5 mg/ml) from each subject was incubated with endothelial cells for 48 h prior to assessing Kir2.1 function via whole-cell patch clamp electrophysiology (representative recordings are shown in Figure 2(B)). Similar to our previous observations using a diet-induced mouse model of obesity, VAT from obese human subjects robustly blunted endothelial Kir current densities, whereas SAT from the same obese human subjects had no effect compared with untreated cells (Figure 2(B,C)). To determine whether inhibiting adipose lipolysis restored endothelial Kir2.1 function in VAT-treated cells, SAT and VAT were first treated with NG-497 (500 nM), a small molecule inhibitor specific to human ATGL (Atglistatin has no effect on human cells or tissue) [32,33], for 2 h prior to assessing endothelial Kir2.1 function. The selected dose of 500 nM represents the lowest IC_50_ of NG-497 that was shown to induce significant inhibition of FA release from human-derived adipocytes in culture, albeit in the presence of another TG hydrolase inhibitor [33]. Therefore, we began our studies with 500 nM NG-497 and, based on our results, maintained this dose throughout the study. NG-497-treated SAT did not significantly influence endothelial Kir2.1 function as compared to vehicle-treated control SAT (Figure 2(B,C)). In contrast, endothelial cells exposed to VAT treated with NG-497 demonstrated a significant recovery in Kir current density, suggesting that the inhibition of lipolysis and, therefore, the generation of FAs specifically from VAT may underlie endothelial Kir2.1 impairment in humans with obesity (Figure 2(B,C)).

Together, the present findings, generated from a mouse model of diet-induced obesity and adipose tissue from obese humans, suggest that inhibiting VAT lipolysis prevents the impairment of endothelial Kir2.1, further supporting the role of VAT-derived FAs in governing Kir2.1 dysfunction in obesity.

### VAT from obese mice and humans does not significantly affect Kir2.1 or CD36 expression

**Figure 3.**
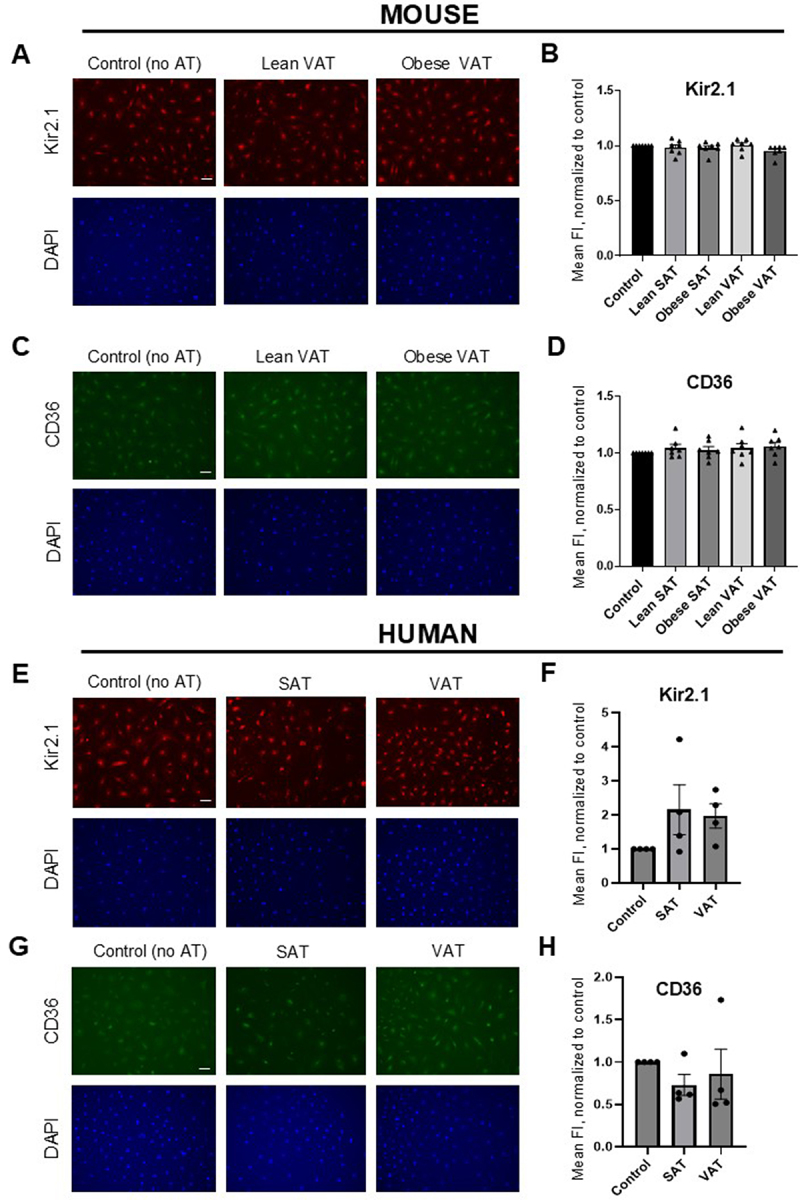
VAT does not affect endothelial Kir2.1 or CD36 expression. HAMECs were incubated en bloc (5 mg/ml) with adipose tissue from lean or obese mice or human subjects with obesity for 48 hours before assessing expression of Kir2.1 or CD36 using immunocytochemistry. HAMECs that were not exposed to adipose served as a control (no AT). A) representative fluorescent microscopy images (10x) of HAMECs showing expression of Kir2.1 after exposure to VAT from lean or obese mice (top). Nuclei were stained with DAPI (bottom). The scale bar is 20 μm. B) normalized group data reveal no differences in mean fluorescence intensity (FI) as determined by a Kruskal-wallis test (*p* = 0.1105) (*n* = 7). C) representative fluorescent microscopy images (10x) of HAMECs showing expression of CD36 after exposure to VAT from lean or obese mice (top). Nuclei were stained with DAPI (bottom). The scale bar is 20 μm. D) normalized group data reveal no differences in mean FI as determined by a Kruskal-wallis test (*p* = 0.4834) (*n* = 7). E) representative fluorescent microscopy images (10x) of HAMECs showing expression of Kir2.1 after exposure to SAT or VAT from human subjects (top). Nuclei were stained with DAPI (bottom). The scale bar is 20 μm. F) normalized group data reveal no differences in mean FI as determined by a Kruskal-wallis test (*p* = 0.0866) (*n* = 4). G) representative fluorescent microscopy images (10x) of HAMECs showing expression of CD36 after exposure to SAT or VAT from human subjects (top). Nuclei were stained with DAPI (bottom). The scale bar is 20 μm. H) normalized group data reveal no differences in mean FI as determined by a Kruskal-wallis test (*p* = 0.4121) (*n* = 4).

Our previous studies revealed that obesity does not influence the expression of Kir2.1 in the VAT artery endothelium [27]. We further showed that adipose tissue also had no effect on the expression of endothelial Kir2.1 or CD36, regardless of depot type (SAT vs. VAT) or source (lean vs. obese mouse) [19]. To better gauge whether VAT affects membrane expression of the target proteins, we performed immunocytochemistry (ICC) using antibodies targeting extracellular epitopes of Kir2.1 and CD36 in unpermeabilized endothelial cells, followed by immunofluorescence imaging. Antibody validation for this approach was first confirmed for both Kir2.1 and CD36 by assessing the specificity of the secondary antibody (no primary antibody in the presence of the protein target; Figure S1A, B) and using siRNA knockdown to assess the specificity of the primary antibody in the absence of the protein target (Figure S1C, D). No significant differences in endothelial Kir2.1 (Figure 3(A,B)) or CD36 (Figure 3(C,D)) were detected when comparing cells treated with VAT or SAT (representative images for SAT-treated endothelial cells are shown in Figure S2) from lean or obese mice to control and untreated cells, thereby supporting our original findings. To determine whether these findings translate to human obesity, we incubated endothelial cells with VAT or SAT from the same human subjects presented in Figure 2. While neither SAT nor VAT from the four subjects with obesity significantly altered the expression of Kir2.1 (Figure 3(E,F)) or CD36 (Figure 3(G,H)) compared to untreated control cells, large variances and trends in the data suggest that differences may be present in a larger sample size. This appears to be especially true for endothelial CD36, where removal of an outlier subject resulted in a significant decrease in CD36 expression in cells exposed to SAT or VAT (Kruskal Wallis test, *p* = 0.0464). However, no apparent differences were observed when comparing normalized SAT and VAT mean FI values for Kir2.1 (*p* = 0.8857 using a Mann Whitney test; Figure 3(F)) or CD36 (*p* > 0.9999 using a Mann Whitney test; Figure 3(H)), which further supports that VAT does not specifically induce differences in the expression of Kir2.1 or CD36 in obesity. When considering the functional impact of VAT on Kir2.1 and the critical role of CD36, these findings suggest that VAT induces events that render Kir2.1 dysfunctional without differentially influencing the expression of these proteins.

### Inhibition of lipolysis in VAT from obese mice and humans significantly blunts fatty acid uptake in endothelial cells

Based on our recent findings showing that VAT from obese mice induces an increase in FA uptake, and our current findings revealing that the inhibition of lipolysis in VAT from obese mice and humans restores Kir2.1 function, we next aimed to determine if i) exogenous FA similarly induces an increase in FA uptake, revealing a possible FA-induced FA uptake mechanism, and ii) inhibition of VAT lipolysis similarly prevents endothelial FA uptake, as we recently observed in endothelial cells lacking CD36 [19]. This was accomplished by imaging internalized fluorescent FA analogs following exposure of endothelial cells to either PA or adipose tissue, as previously described [19,31].

**Figure 4.**
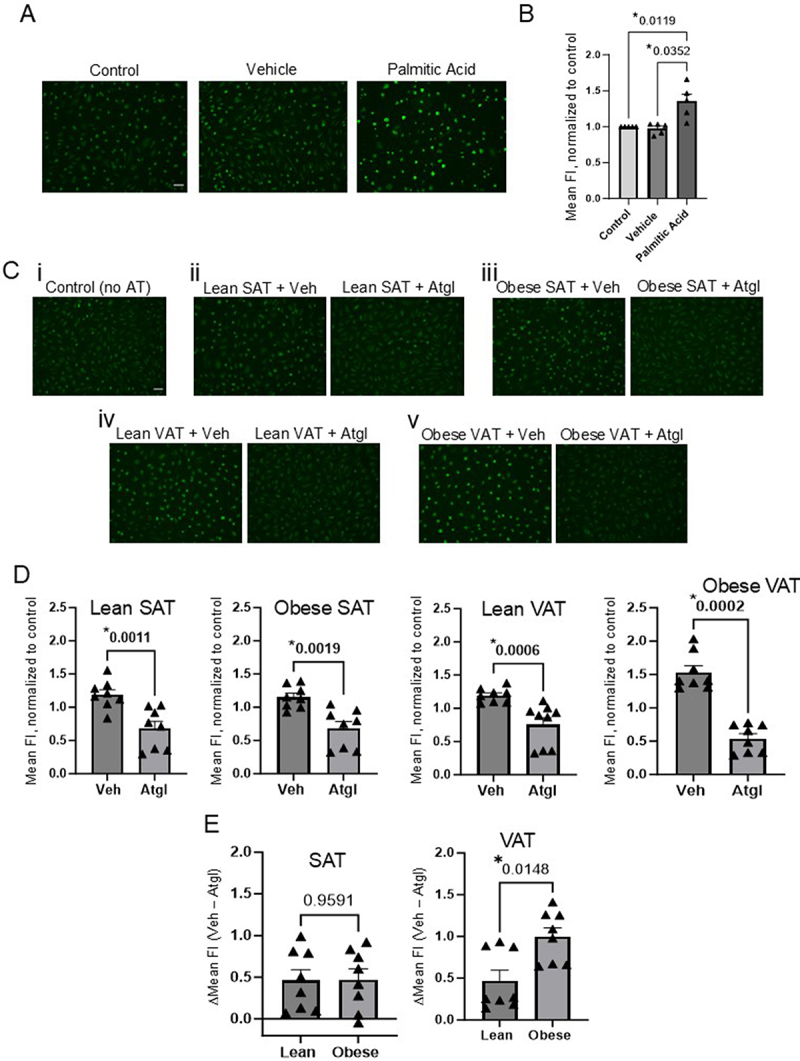
Inhibition of lipolysis in VAT from obese mice significantly blunts fatty acid uptake in endothelial cells. HAMECs were exposed to either 250 μM palmitic acid (PA) or adipose tissue from lean or obese mice (en bloc; 5 mg/ml) for 48 hours prior to assessing FA uptake. Untreated HAMECs, HAMECs that received BSA sans FA (vehicle control; veh), and cells that were not exposed to adipose (no AT) served as respective controls. Adipose ATGL was inhibited by incubating adipose tissue with 20 μM Atglistatin for 2 hours prior to exposing HAMECs to adipose tissue. A) Representative fluorescent microscopy images (10x) show fluorescent FA uptake in HAMECs following incubation with PA. The scale bar is 20 μm. B) Mean fluorescence intensity (FI) group data was normalized to the untreated control and significant differences were identified using a Kruskal-Wallis test followed by a Dunn’s test post hoc correction. Five independent experiments were performed. Significant differences are denoted by (*). C) Representative fluorescent microscopy images (10x) show fluorescent FA uptake in (i) control (no AT) cells and cells exposed to (ii) SAT from lean mice pretreated with vehicle (veh) or Atglistatin, (iii) SAT from obese mice pretreated with veh or Atglistatin, (iv) VAT from lean mice pretreated with vehicle (veh) or Atglistatin, and (v) VAT from obese mice pretreated with veh or Atglistatin. The scale bar is 20 μm. D) The mean FI was normalized to untreated controls (no AT) before assessing the effects of inhibiting lipolysis with Atglistatin in SAT and VAT from lean or obese mice. Significant differences (*) in FA uptake in HAMECs exposed to adipose pretreated with veh or Atglistatin within respective adipose and diet groups were identified using a Mann-Whitney test (*n* = 8). E) Normalized mean FI deltas (veh FI – Atglistatin FI), generated using the veh and Atglistatin FI values from each independent adipose sample, reveal stark differences in endothelial FA uptake after lipolysis was inhibited in SAT vs. VAT. Significant differences (*) were identified using a Mann-Whitney test.

Figure 4(A) shows representative fluorescence images comparing FA uptake in PA-treated cells to that of untreated cells and FA-free BSA vehicle-treated cells. As observed in our previous study using VAT from obese mice, PA induced a significant increase in FA uptake compared to the control condition (Figure 4(B)). To further assess the role of VAT-derived FAs in mediating an increase in endothelial FA uptake, we treated SAT and VAT from lean or obese mice with Atglistatin prior to en bloc exposure of endothelial cells to adipose tissue. As shown in representative images (Figure 4(C)), control cells (i) that did not receive adipose treatment exhibited a basal level of FA uptake that was not significantly altered by either SAT from lean (ii) or obese (iii) mice or VAT from lean mice (iv) (average increase in fluorescence was ~ 15–20% in these groups compared to control cells, Figure 4(D)). In contrast, in accordance with our previous findings, VAT from obese mice (v) induced an average increase in fluorescence of more than 53% compared to control cells (Figure 4(D)). Endothelial cells exposed to adipose tissue treated with Atglistatin revealed significant decreases in FA uptake independent of adipose depot (i.e. SAT vs. VAT) or source (i.e. lean vs. obese) compared to adipose tissue from the same mice treated with vehicle control, indicating that the inhibition of lipolysis in any of these adipose depots can effectively blunt endothelial FA uptake (Figure 4(C,D)). However, the extent of the observed reduction in fluorescence among the Atglistatin-treated groups compared with the matched vehicle-treated adipose groups was significantly different. Specifically, endothelial cells exposed to Atglistatin-treated VAT from lean mice or SAT from lean or obese mice exhibited a similar reduction in average fluorescence of approximately 40%, whereas cells exposed to Atglistatin-treated VAT from obese mice exhibited a more profound average decrease in fluorescence of approximately 65% (Figure 4(D)). The effect of Atglistatin on VAT-mediated increases in FA uptake was further highlighted when comparing the vehicle minus Atglistatin deltas in SAT and VAT of lean and obese mice. The normalized values generated for each treatment group (i.e. vehicle vs. Atglistatin) were used to generate a fluorescence intensity value representing the impact of Atglistatin-induced inhibition on endothelial FA uptake within each individual mouse adipose tissue sample. This analysis revealed that endothelial FA uptake was similarly influenced by Atglistatin-treated SAT in both lean and obese mice. In contrast, there was a significant Atglistatin-mediated effect on endothelial FA uptake in cells exposed to VAT from obese mice compared to cells exposed to VAT from lean controls (Figure 4(E)), thereby supporting a VAT-induced increase in endothelial FA uptake in obese mice that was significantly reduced by inhibiting lipolysis.

**Figure 5.**
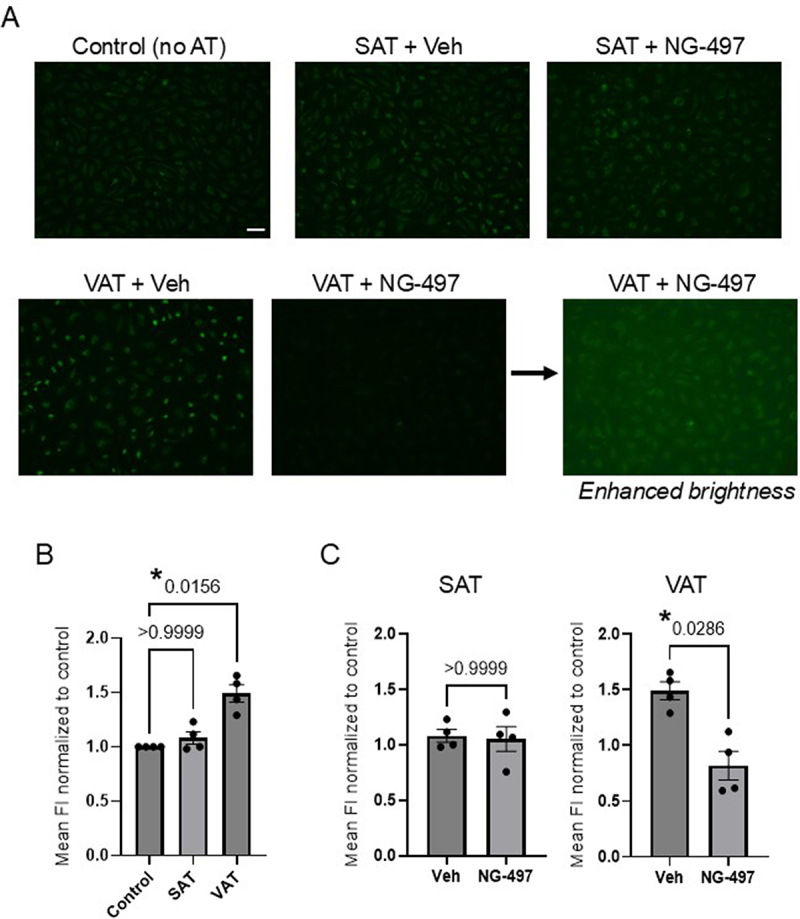
Inhibition of lipolysis in VAT from humans with obesity significantly blunts fatty acid uptake in endothelial cells. HAMECs were exposed to adipose tissue from human subjects with obesity (en bloc; 5 mg/ml) for 48 hours prior to assessing FA uptake. Cells that were not exposed to adipose (no AT) served as control. Adipose ATGL was inhibited by incubating adipose tissue with 500 nM NG-497 for 2 hours prior to exposing HAMECs to adipose tissue. A) Representative fluorescent microscopy images (10x) show fluorescent FA uptake in HAMECs following incubation with SAT or VAT with and without (DMSO vehicle control; veh) NG-497 pretreatment. The scale bar is 20 μm. As the representative image for NG-497-treated VAT shows little fluorescence, the same image is shown again with enhanced brightness to reveal the presence of cells in this field of view. B) Mean fluorescence intensity (FI) Group data comparing the effects of SAT and VAT on FA uptake as compared to control. Data was normalized to the untreated control and significant differences were identified using a Kruskal-Wallis test (*p* = 0.0026) followed by a Dunn’s test post hoc correction. Significant differences are denoted by (*). C) Mean FI group data showing the effects of treating SAT and VAT with NG-497 on endothelial FA uptake. Significant differences (*) were identified using a Mann-Whitney test.

To determine whether these findings translate to human obesity, we incubated endothelial cells with VAT or SAT from the same human subjects presented in Figures 2 and 3. Similar to our recent findings comparing VAT and SAT of obese mice [19], VAT induced a significant uptake of FAs in endothelial cells compared to the control, whereas cells exposed to SAT from the same subjects showed no significant difference (Figure 5(A,B)). Inhibition of SAT ATGL with NG-497 had no effect on FA uptake in endothelial cells compared to cells exposed to vehicle-treated SAT (Figure 5(A,C)). In contrast, inhibiting VAT ATGL resulted in a drastic decrease in endothelial cell FA uptake compared to the respective vehicle-treated control VAT (Figure 5(A,C)), a finding that is in line with our observations using a mouse model of diet-induced obesity in the present study (Figure 4).

Taken together, the present findings, generated from a mouse model of diet-induced obesity and adipose tissue from humans with obesity, suggest that VAT-derived FA may promote endothelial FA uptake as an underlying mechanism governing obesity-induced endothelial dysfunction.

## Discussion

Visceral obesity is now recognized as a significant risk factor for cardiovascular disease, with the accumulation of abdominal VAT being strongly correlated with disease progression [1–4]. Given that adipose tissue is now recognized as a prominent endocrine and paracrine tissue with the capacity to govern vascular function [34], understanding the role of VAT in mediating obesity-induced endothelial dysfunction may unveil new targets to combat disease progression. Here, we expand on our recent findings revealing a putative VAT/CD36/Kir2.1 axis wherein we showed that VAT induced impairment of endothelial Kir2.1 in a CD36-dependent fashion [19]. Specifically, these findings linked VAT to the upregulation of CD36, a scavenger receptor and FA-translocase well-established in cardiovascular disease progression [23], as an upstream event driving the dysfunction of Kir2.1 in obesity [27]. However, how VAT governs this axis remains unresolved.

In the present study, we further probed the role of VAT in mediating the impairment of endothelial Kir2.1 by assessing the role of VAT as a potential paracrine tissue with a focus on VAT-derived FAs. Our hypothesis that the inhibition of lipolysis in VAT from obese mice and human subjects prevents Kir2.1 impairment is based on the following lines of evidence: first, the release of FAs from VAT is a major contributor to insulin resistance and cardiovascular disease [7,12–14]. Accordingly, FAs are well-established to induce endothelial dysfunction when in excess [15]. Furthermore, our initial findings linked CD36, a major endothelial FA translocase, to both impairment of Kir2.1 channels and enhanced endothelial FA uptake following VAT exposure [19]. Finally, Kir channels were previously shown to be inhibited by several classes of FA derivatives [25,26]. Therefore, we first aimed to determine whether the exogenous addition of a physiologically abundant FA, palmitic acid (PA), recapitulated our original findings showing that VAT impaired endothelial Kir2.1 via CD36. Indeed, PA blunted Kir2.1 function, an effect that was prevented by downregulating endothelial CD36, thereby serving as the first indication that VAT-derived FAs may play a role. To determine whether VAT-derived FAs are potential mediators of the impairment of endothelial Kir2.1, we prevented the rate-limiting step in lipolysis by inhibiting ATGL in VAT from obese mice and humans prior to exposing endothelial cells to VAT in vitro. This approach prevented VAT-induced impairment of endothelial Kir2.1 function and supports a role for VAT-derived FAs as paracrine mediators of Kir2.1 function.

### Potential role of VAT-derived FAs in impairing Kir2.1 function

We recently showed that VAT from obese mice did not influence total Kir2.1 or CD36 expression in endothelial cells, as assessed by western blotting [19]. However, this approach does not reveal membrane expression which may be differentially regulated independent of total protein. Therefore, we performed ICC on nonpermeabilized cells with antibodies to Kir2.1 and CD36 that targeted extracellular epitopes to better observe potential alterations in expression at the membrane following exposure to adipose tissue. Our findings assessing expression in this way further support that VAT does not induce a change in Kir2.1 or CD36 expression and, when taken together with observed impairments in Kir2.1 function and enhanced fatty acid uptake in response to VAT from obese mice and humans, prompts speculation of functional alterations to each protein.

The role of FAs in governing endothelial dysfunction has been well investigated in previous studies, pointing to the induction of oxidative stress as a major mechanism [15]. FAs have been shown to promote the formation of reactive oxygen species (ROS) in many cell types, including endothelial cells [35–37]. Elevated ROS production damages a myriad of cellular components, possibly including Kir2.1 and/or essential regulators of the channel. We and others have shown that the endothelial glycocalyx, a sugar-rich extracellular extension into the vessel lumen, is degraded under obesogenic conditions [16,27,38]. This is noteworthy because of i) the established sensitivity of the glycocalyx to ROS-mediated disruption [39,40] and ii) our previous finding that the endothelial glycocalyx positively regulates Kir2.1 activity [27]. Therefore, FA-dependent increases in ROS that subsequently disrupt the glycocalyx may indirectly render Kir2.1 channels nonfunctional in obesity; however, whether VAT explicitly disrupts the endothelial glycocalyx in obesity remains to be determined. In addition, ROS may have the capacity to directly inhibit Kir2.1 and while this has not been specifically shown for Kir2.1, it contains relevant residues as other Kir channels that were shown to be inhibited under oxidative conditions [41].

While FAs and ROS have a clear link to endothelial dysfunction, Kir2.1 channels are also well known to be sensitive to the lipid milieu with distinct lipids that positively regulate channel function (e.g. PIP2) and others inhibiting the channel (e.g. cholesterol) [42–44]. Different classes of FA derivatives have also been shown to inhibit Kir2.1 channels, including long-chain fatty acyl CoA esters and ceramides, and direct and indirect mechanisms of inhibition have been proposed for these metabolites [25,26]. Based on the CD36-dependency of Kir2.1 inhibition in the presence of exogenous PA and VAT from obese mice, it is possible that the internalization and subsequent conversion of FAs to one of these lipid metabolites (i.e. ceramides, long-chain esters) drives endothelial Kir2.1 dysfunction. Future studies that prevent the formation of FA derivatives, such as inhibiting the enzymes that convert FAs to ceramide, are warranted to determine if this restores Kir2.1 function in the presence of VAT and FAs. In addition, reordering of the lipid membrane and/or displacement of PIP2, events that may also affect Kir2 channels [42,45–47], are plausible events following CD36-mediated uptake of FAs.

Determining whether one or more of these mechanisms underlie VAT- and FA-induced impairment of Kir2.1 represents a significant step forward in revealing suitable targets to prevent Kir2.1 impairment and, therefore, endothelial dysfunction in obesity. Furthermore, recent findings suggest that Kir2.1 regulation by lipids may be cell type-dependent in the vasculature [48]. This evidence emphasizes the role of distinct adipose tissue depots in contributing to the differential regulation of Kir2.1 and other vascular ion channels, perhaps as mediated by paracrine FAs, under physiological and disease conditions.

### Therapeutic implications of deciphering a putative VAT/FA/CD36/Kir2.1 axis in obesity

The breakdown of triglycerides that ultimately results in free FAs is a multienzyme process, the rate limiting step of which is the ATGL-mediated separation of the initial FA from glycerol. Previous efforts by Mayer et al. (2013) and Grabner et al. (2022) to identify suitable inhibitors of lipolysis resulted in two distinct small-molecule inhibitors separately catered to mouse (i.e. Atglistatin) and human (i.e. NG-497) ATGL [32,33]. These studies demonstrated that each inhibitor drastically blunted lipolysis in vitro, and that Atglistatin significantly reduced circulating free FAs in mice in vivo. In the present study, we revealed that inhibiting lipolysis by targeting ATGL in VAT from obese mice and humans with obesity with respective inhibitors ex vivo prevented VAT-induced impairment of Kir2.1. Moreover, Atglistatin or NG-497 treatment in mouse and human VAT significantly reduced endothelial FA uptake compared to control VAT from the same mice and humans treated with vehicle only. The latter finding in the present study, coupled with observations of a PA-induced increase in endothelial FA uptake, recapitulates our initial observations of endothelial CD36 downregulation. Together, these findings point to a role for a CD36-mediated FA uptake mechanism initiated by VAT that results in the impairment of Kir2.1, prompting further investigation into a VAT/FA/CD36/Kir2.1 axis as a contributor to obesity-endothelial dysfunction.

Elevated FA mobilization from adipose triglyceride stores in obesity is an established contributor to insulin resistance with links to adipose tissue inflammation as a driving factor [49,50]. Previous studies have shown that inhibiting adipose tissue lipolysis, including the targeting of ATGL with Atglistatin, in vivo restores systemic insulin sensitivity in rodent models of obesity and diabetes [51–53]. Importantly, our findings also allude to the potential therapeutic benefit of inhibiting VAT lipolysis as a means to combat the progression of cardiovascular disease, potentially through restoring endothelial function. Endothelial Kir2.1 channels are critical regulators of vasomotor tone, playing essential roles in promoting vasodilation in response to mechanical and receptor-mediated cues [21,22]. As such, loss of endothelial Kir2.1 function results in blunted endothelium-dependent dilatory responses [16,27–29,44], a hallmark of endothelial dysfunction, thereby serving as a potential precursor to advanced disease. Indeed, we recently showed that blunted expression of Kir2.1 resulted in exacerbated atherosclerotic lesion formation in a mouse model of dyslipidemia [44], further supporting an important role for this channel in maintaining vascular homeostasis. While the present study did not directly assess endothelial function, our findings suggest that the pharmacological targeting of adipose lipolysis, specifically through the inhibition of ATGL, may be a promising avenue to restoring endothelial function in obesity by preventing VAT-induced impairment of endothelial Kir2.1.

### Limitations and other considerations

The present in vitro study sheds light on a novel VAT/FA/CD36/Kir2.1 axis in obesity-induced endothelial dysfunction, although we have yet to establish a concrete role for this axis in vivo. Obesity is a complex, multifactorial metabolic condition, and many variables may play a role. An important point to note in this study is that despite inhibiting ATGL and presumably blunting FA release from VAT as previously shown [32], this was not tested directly in our study. Therefore, it is possible that inhibiting ATGL affects a myriad of signaling processes linked to, for instance, adipose-derived ROS production and/or adipokine secretion though whether these factors impair endothelial Kir2.1 remains to be determined. Furthermore, ATGL is expressed in resident macrophages [54,55], which would be present in our en bloc culture method, and may represent the effective cell type leading to Kir2.1 impairment perhaps through inflammatory signaling. Cell-type specific ablation of ATGL and immune cell depletion studies should be considered in parsing the role of adipocytes, resident macrophages, and distinct adipose-derived mediators that drive endothelial Kir2.1 dysfunction.

Our findings also indicate that a decrease in Kir2.1 function, as opposed to a decrease in channel expression, underlies VAT-induced impairment of Kir2.1. However, while the whole-cell patch clamp technique is the standard approach to assess Kir channel function in cells because it allows for easy comparison of large inwardly rectifying currents, it does not directly inform on channel open probability. Assessing Kir2.1 function in relevant single channel configurations (e.g. inside-out, outside-out patches) in endothelial cells and expression systems would directly address if VAT and FAs decrease the open probability of the channel. Such studies, specifically in the inside-out configuration, would also determine if VAT-derived FAs have the capacity to directly and acutely inhibit Kir2.1 through binding intracellular residues, an effect that would support the dependence on CD36-mediated FA internalization in intact cells. However, it should also be noted that the influence of VAT-derived FAs on other ion channels and signaling mediators could serve as complementary or even parallel paths to endothelial dysfunction. Other major endothelial ion channels, including Piezo1 and select members of the transient receptor potential (TRP) family of channels (e.g. TRPM2/4) have each been shown to be modulated by saturated FAs in ways that could promote endothelial dysfunction. Similar to endothelial Kir2.1 channels, activation of Piezo1 channels, which were shown to be inhibited by FAs [56], has also been linked to nitric oxide production and endothelium-dependent vasodilation [57]. In contrast, FA-induced increases in TRPM4 expression and activation of TRPM2 channels, likely in response to FA-induced oxidative stress [58], were each shown to result in Ca^2+^ overload, an event that promotes cell death and endothelial dysfunction [59,60]. As we have yet to determine if specifically preventing VAT-induced impairment of endothelial Kir2.1 restores endothelial function in intact arteries, these ion channels may very well be part of convergent processes that collectively blunt the production of key vasodilatory mediators, ultimately resulting in endothelial dysfunction.

While the human data revealed a robust influence on endothelial Kir2.1 function and FA uptake, data from only four subjects with obesity are presented. This small sample size does not yet allow for stratifying the data based on demographic/clinical values or differences in medication, analyses that will certainly expand our understanding of obesity-induced endothelial dysfunction in this population. Finally, while SAT serves as an appropriate within-subject control for the effects of VAT on endothelial cell function, lean “healthy” controls were not available in human studies because of the source of biopsy collection (i.e. bariatric surgery). Therefore, in the absence of adipose from individuals without obesity, interpretation of our findings solely using this source of human adipose tissue has limited generalizability as we cannot yet confirm that VAT from lean individuals has no effect on endothelial Kir2.1 and endothelial FA uptake. Future translational studies that include non-obese individuals undergoing surgeries that afford access to relevant adipose tissue (e.g. hernia repair) may offer increased rigor when evaluating the role of VAT in obesity-induced endothelial dysfunction. However, caution should be exercised when considering this population as lean and/or “healthy” controls.

## Conclusions

Our findings advance our understanding of the role of VAT in mediating endothelial cell dysfunction and highlight the impact of lipolysis and FAs on Kir2.1 channels in obesity. The present findings, generated from a mouse model of diet-induced obesity and adipose tissue from humans with obesity, suggest that VAT-derived FAs promote endothelial Kir2.1 dysfunction perhaps following elevated FA uptake as an underlying mechanism governing obesity-induced endothelial dysfunction. Future studies should further investigate the potential VAT/FA/CD36/Kir2.1 axis in the context of obesity-induced endothelial dysfunction.

## Supplementary Material

Figure S1.JPG

Table S1.JPG

Table S2.JPG

Figure S2.JPG

## Data Availability

The authors confirm that the data supporting the findings of this study are available in the article and its supplementary materials. Data and materials supporting the results or analyses in this study will be made available upon reasonable request.
