# Supplementary figures and images for: Inhibition of lipolysis in visceral adipose tissue from obese mice and humans prevents impairment of endothelial Kir2.1 channels
Source: Channels (Austin). 2025 Sep 25;19(1):2564651. doi: 10.1080/19336950.2025.2564651 (PMC12477883; doi:10.1080/19336950.2025.2564651)

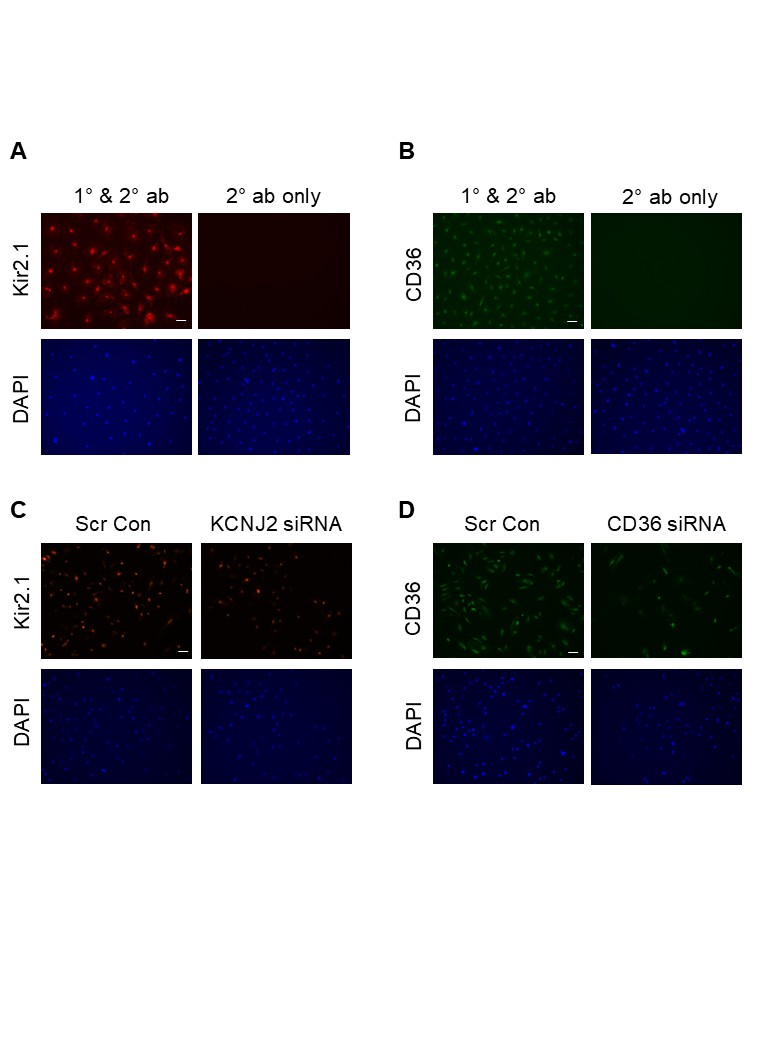

Supplement: Figure S1.JPG [file KCHL_A_2564651_SM4989.jpg]

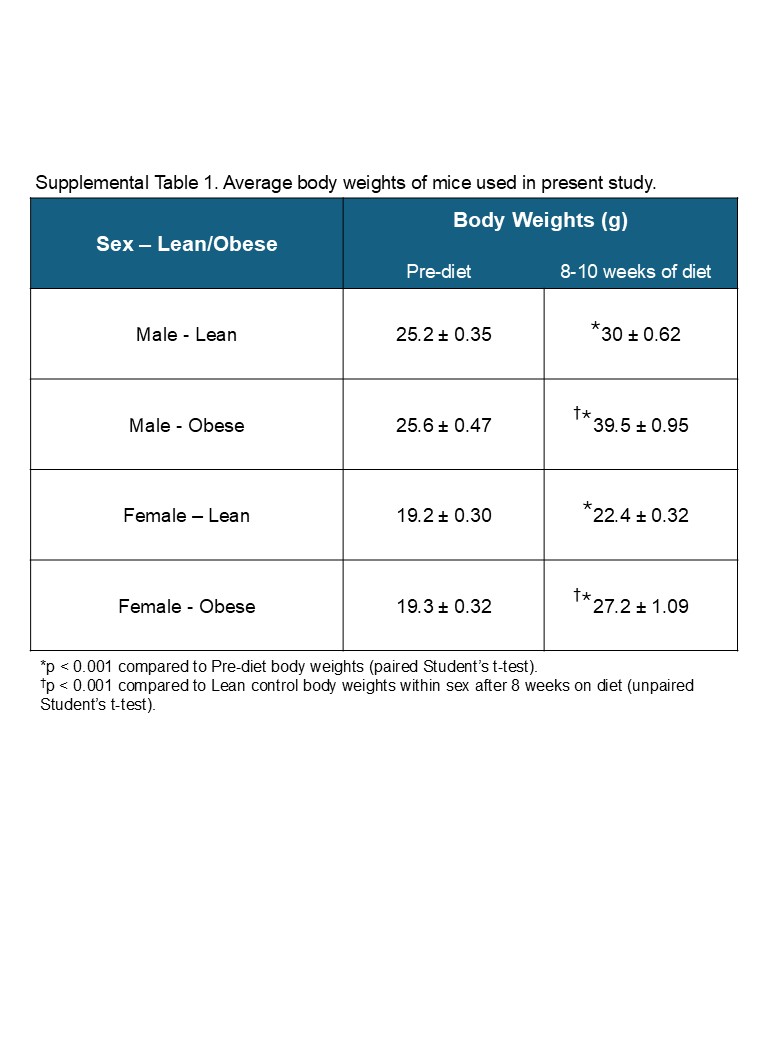

Supplement: Table S1.JPG [file KCHL_A_2564651_SM4988.jpg]

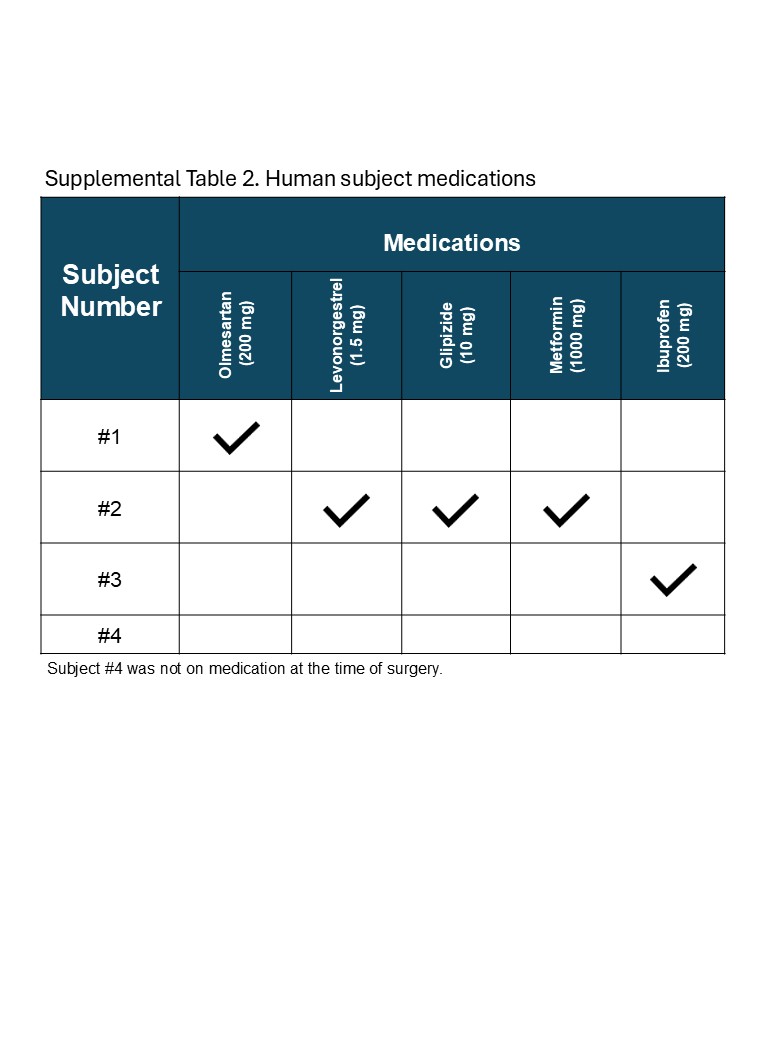

Supplement: Table S2.JPG [file KCHL_A_2564651_SM4987.jpg]

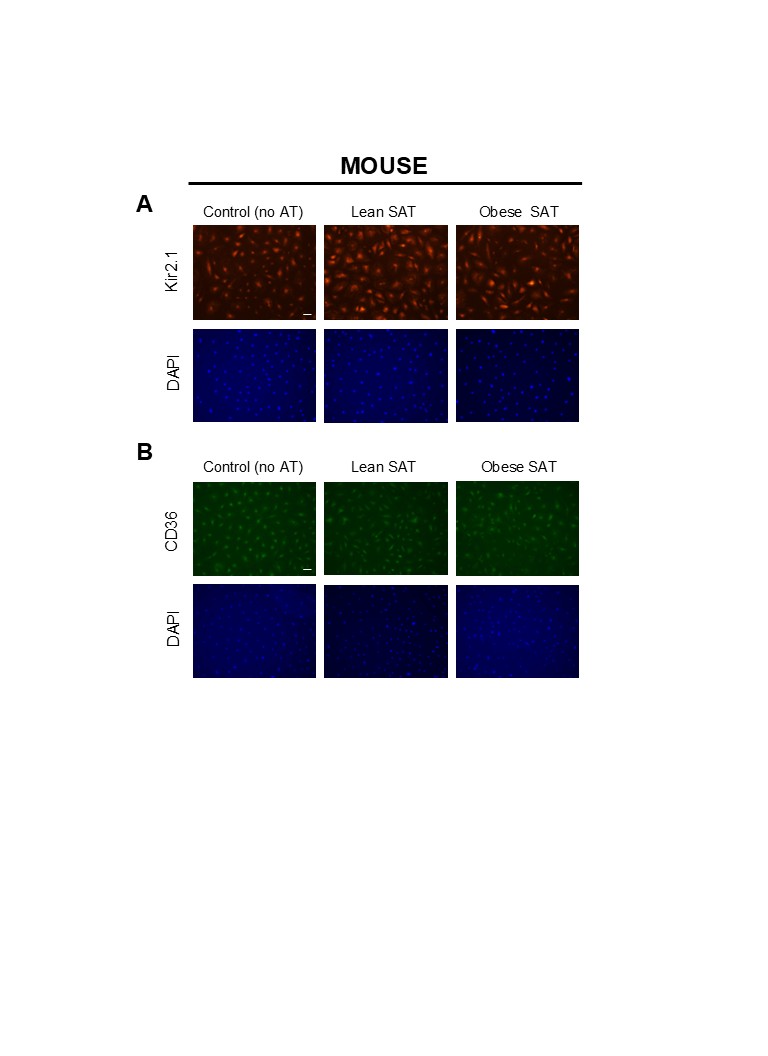

Supplement: Figure S2.JPG [file KCHL_A_2564651_SM4985.jpg]
